# Improving the quality of neonatal health care in Ethiopia: a systematic review

**DOI:** 10.3389/fmed.2024.1293473

**Published:** 2024-05-22

**Authors:** Demeke Mesfin Belay, Daniel Erku, Wubet Alebachew Bayih, Yohannes Tesfahun Kassie, Binyam Minuye Birhane, Yibeltal Assefa

**Affiliations:** ^1^College of Health Sciences, Debre Tabor University, Debre Tabor, Ethiopia; ^2^Menzies School of Health Research, Charles Darwin University, Darwin, NT, Australia; ^3^School of Public Health, Faculty of Medicine, The University of Queensland, Herston, QLD, Australia; ^4^Centre for Applied Health Economics, Griffith University, Nathan, QLD, Australia; ^5^Menzies Health Institute Queensland, Griffith University, Gold Coast, QLD, Australia; ^6^ Addis Consortium for Health Economics and Outcomes Research (AnCHOR); ^7^Department of Epidemiology and Preventive Medicine, School of Public Health and Preventive Medicine, Faculty of Medicine, Nursing and Health Sciences, Monash University, Melbourne, VIC, Australia; ^8^School of Public Health, University of Technology Sydney, Sydney, NSW, Australia

**Keywords:** Ethiopia, neonatal health, quality of care, quality improvement, systematic review

## Abstract

**Background:**

Ensuring high-quality healthcare for newborns is essential for improving their chances of survival within Ethiopia's healthcare system. Although various intervention approaches have been implemented, neonatal mortality rates remain stable. Therefore, the present review seeks to identify initiatives for enhancing healthcare quality, their effects on neonatal wellbeing, and the factors hindering or supporting these Quality Improvement (QI) efforts' success in Ethiopia.

**Methods:**

We searched for original research studies up to June 23, 2023, using PubMed/Medline, WHO-Global Health Library, Cochrane, Clinical Trials.gov, and Hinari. After selecting eligible studies, we assessed their quality using a mixed-method appraisal tool. Quality of care refers to how healthcare services effectively improve desired outcomes for individuals and patient populations. It encompasses vital principles such as safety, effectiveness, timeliness, efficiency, equity, and patient-centeredness.

**Results:**

We found 3,027 publication records and included 13 studies during our search. All these interventions primarily aimed to provide safe healthcare, with a strong focus on Domain One, which deals with the evidence-based routine upkeep and handling of complications, and Domain Seven, which revolves around ensuring staff competency, emerged as a frequent target for intervention. Many interventions aimed at improving quality also concentrate on essential quality measure elements such as processes, focusing on the activities that occur during care delivery, and quality planning, involving distributing resources, such as basic medicine and equipment, and improving infrastructure. Moreover, little about the facilitators and barriers to QI interventions is investigated.

**Conclusions:**

This review highlights the significance of introducing QI initiatives in Ethiopia, enhancing the healthcare system's capabilities, engaging the community, offering financial incentives, and leveraging mobile health technologies. Implementing QI interventions in Ethiopia poses difficulties due to resource constraints, insufficient infrastructure, and medical equipment and supplies shortages. It necessitates persistent endeavors to improve neonatal care quality, involving ongoing training, infrastructure enhancement, the establishment of standardized protocols, and continuous outcome monitoring. These efforts are crucial to achieving the optimal outcomes for newborns and their families.

## Background

The initial month of a child's life is the most crucial period for their survival (1). In 2020, there were 2.4 million newborn fatalities worldwide. Neonatal mortality rates are more significant in low-income nations compared to high-income ones. Sub-Saharan Africa has the highest neonatal mortality rate globally, accounting for 43% of neonatal deaths worldwide (2).

The 2020 World Health Organization (WHO) report highlighted Ethiopia's high neonatal mortality rate of 97 deaths per 1,000 live births, placing it among countries with the highest rates globally. Nations with such high mortality rates, including Ethiopia, faced a significantly higher risk of neonatal mortality compared to those with the lowest rates (2). The subpar quality of care for newborns is a significant factor contributing to neonatal mortality rates (3, 4). To address this issue, the Sustainable Development Goals (SDGs) aim to reduce global neonatal mortality rates to 12 per 1,000 live births and stillbirth rates to 9 per 1,000 live births by 2030. Achieving these goals requires healthcare systems to prioritize quality of care as a fundamental principle and commit to improving neonatal health outcomes (5–8). This involves establishing standardized guidelines for neonatal care (9) and implementing legal directives in routine healthcare practices (10–12). The WHO has set standards to enhance the quality of maternal and neonatal care (13). At the same time, the global Every Newborn Action Plan (ENAP) focuses on improving newborn care through QI initiatives (12). Quality healthcare encompasses safe, effective, timely, efficient, equitable, and integrated care tailored to patients' needs (14, 15).

In 2015, Ethiopia established the Ethiopian National Quality Strategy (NQS) with a specific focus on improving newborn health, aligning it with the country's Health Sector Transformation Plan (HSTP) (16, 17). Consequently, Ethiopia embraced WHO standards and introduced the Health Sector Transformation in Quality (HSTQ) initiative to enhance the quality of newborn healthcare within healthcare facilities (16). To align with the evolving NQS, a quality standard was developed for assessing healthcare quality, and the HSTQ serves as the standard document, conforming to WHO neonatal healthcare quality standards and facilitating the implementation of the NQS (13, 16). The NQS identifies three fundamental components of quality: quality planning, QI, and quality control (16). Nevertheless, Ethiopia's healthcare system faces significant challenges, including resource and funding shortages, a scarcity of medical personnel, heavy reliance on external aid, limited infrastructure, and the complexities of serving a geographically diverse population (18). These conditions undermine national efforts to enhance the quality of healthcare, resulting in increased rates of newborn morbidity and mortality (19).

On a global scale, health policy planners, healthcare providers, and public health researchers have recognized the imperative of delivering high-quality healthcare through effective quality measures (20). Quality interventions encompass the implementation of systematic processes, actions, or measures, whether individually or in combination, aimed at enhancing the quality of care provided to newborns (21, 22). In response to this, the WHO has established eight domains that define the quality of care for improving maternal and newborn healthcare. These domains can serve as a framework for various QI interventions (13). Enhancing the quality of healthcare for newborns has the potential to prevent 531,000 stillbirths and 1.3 million neonatal deaths annually (20).

The Ethiopian healthcare system encounters difficulties in achieving fair access to top-notch care, despite the longstanding commitment of the WHO to ensuring quality care. This review addresses the absence of a systematic evaluation of interventions aimed at improving the quality of neonatal healthcare in Ethiopia. The primary objective of this review is to examine and consolidate the implementation of these interventions, gauge their impact on the quality of neonatal healthcare services and survival rates, and pinpoint the factors that hinder or facilitate these efforts to enhance quality. Additionally, the review seeks to provide insights for policymakers, healthcare providers, stakeholders, and decision-makers regarding the enhancement of quality measures. It will assess interventions, identify areas in need of further research, and contribute to the overall improvement of neonatal healthcare in Ethiopia.

## Methods and materials

### Reporting, protocol, and registration

We reached out to experts in methodological and systematic reviews during the development of this review protocol. The protocol was then prepared and registered with the International Prospective Register of Systematic Reviews (PROSPER). To uphold transparency and the ability to reproduce this review, we followed the standard guidelines outlined in the Preferred Reporting Items for Systematic Review and Meta-analysis (PRISMA) (23) (see Supplementary material).

### Data sources and search strategy

To access available data, we conducted a thorough review of literature from academic databases, project reports, and documents. Initially, we collaborated with librarians to identify appropriate search terms and databases. Subsequently, we developed comprehensive search strategies aimed at identifying relevant studies. Our search spanned multiple databases, including PubMed, Medline, Cochrane, and Hinari. Furthermore, we examined WHO trial registries, such as the International Clinical Trials Registry Platform (ICTRP), the International Standard Randomized Controlled Trial Number (ISRCTN), and ClinicalTrials.gov, to identify both ongoing and completed studies. In addition to these methods, we performed a manual search, which entailed a meticulous examination of cross-references and bibliographies within the selected publications to uncover any additional relevant articles. Furthermore, we expanded our search scope to include gray literature through a Google search. The comprehensive searches commenced on March 25, 2022, and an updated search was conducted on June 23, 2023. We followed the Population, Intervention, Comparison, and Outcome (PICO) search format and employed various Medical Subject Heading (MeSH) search terms, such as quality, quality improvement, quality control, quality planning, quality indicator, interventions, approaches, postnatal care, infant care, perinatal care, child health services, health care, health services, neonatal care, neonatal health, newborn health, maternal-child health service, and Ethiopia, while searching the databases. Boolean operators, such as “AND” and “OR” were utilized to combine search terms. Accordingly, we have conducted search strategies for each database, and the results for the PubMed database are provided in the attached document (Supplementary material).

### Eligibility criteria

In this review, we included both published and unpublished studies without any restrictions on their study period, if they were published in the English language. Unpublished studies were accessed through manual searches or online repositories. To be considered for inclusion in this review, articles needed to contain information regarding the population, intervention, comparison, and outcomes of QI interventions. Additionally, studies that reported neonatal health care QI intervention and were conducted in Ethiopia were included in this review.

**Population:** The eligible populations encompassed systems, organizations, or providers involved in the care of newborns, whether in inpatient or community settings. Additionally, newborns in healthcare facilities were considered part of the population.

**Intervention:** QI interventions were defined as systematic processes or actions designed to address quality gaps and result in measurable improvements in neonatal health services and the health status of the targeted populations or beneficiaries (24).

**Comparison:** The comparison group either received no QI intervention or was subjected to an intervention that did not enhance the quality of care for newborns.

**Outcomes:** Quality of care was defined as the extent to which healthcare services effectively improved desired outcomes for individuals and patient populations. It encompassed the principles of safety, effectiveness, timeliness, efficiency, equity, and patient-centeredness (13). However, commentaries, letters to the editor, and editorials were excluded from consideration in this review.

### Study selection

Initially, the reference manager EndNote version 9 (Thomas Reuters, London) was employed to eliminate duplicate studies. The process of selecting studies involved a sequence of steps, including screening the titles and abstracts, followed by a full-text screening using a standardized tool provided by the Joanna Briggs Institute (JBI). Two authors (DMB and YA) independently reviewed all the titles and abstracts of the studies to identify those that potentially met the inclusion criteria. Any titles and abstracts that could not definitively be included or were excluded after the initial screening were reviewed by two other authors (DE and WAB) to identify any additional eligible studies. Furthermore, another author (BMB) examined the reference lists to identify any other articles that might be relevant. The full-text review was conducted by two authors (YTK and YA), and any discrepancies were resolved by a third author (BMB).

### Data extraction

We created an Excel spreadsheet for data extraction, which was pre-piloted and standardized. This spreadsheet was initially tested on a sample of 10 articles and continuously adjusted as necessary. During the data extraction process for the selected studies, we collected information on several aspects, including the study's author and publication year, study characteristics, specific details about the QI interventions, participant demographics, metrics used to assess QI outcomes, and the findings of the study.

### Quality appraisal and risk of bias assessment

We assessed the methodological quality of each study using the Mixed Methods Appraisal Tool (MMAT) (25). Two independent authors conducted the evaluation of risk of bias and quality appraisal, with any discrepancies resolved through discussion with a third author. The MMAT is suitable for this review as it allows for the simultaneous assessment of methodological quality across various study types, including quantitative studies, randomized control trials, non-randomized control trials, and mixed-method studies (26). Different criteria for methodological quality appraisal were applied to different types of studies. Consequently, separate assessment tools were used to gauge the methodological quality of randomized and non-randomized studies. It's worth noting that not all the studies and reports included provided sufficient information for a comprehensive quality assessment using the MMAT. However, it's important to clarify that quality scores were not utilized to include or exclude studies; rather, they were employed to describe the quality of the available evidence as part of the review's mapping component (Table 1).

**Table 1:** Quality appraisal using mixed method appraisal tool, Ethiopia, 2023.

**References**	**Methodological quality criteria**					
	**Non-randomized controlled trials**					**Score**
	**Has randomization been carried out correctly and in a suitable manner?**	**Do the groups exhibit comparability in terms of their initial characteristics?**	**Is there comprehensive and unaltered outcome data available?**	**Are the individuals responsible for assessing the outcomes unaware of the specific intervention that was administered?**	**Did the participants follow the prescribed intervention as instructed?**	
Hailemeskel et al. (27)	Yes	No	Yes	No	Yes	Three
Hagaman et al. (28)	No	Yes	Yes	No	Yes	Three
	**Other observational studies**					
	**Does the chosen sampling method align with the research question being addressed?**	**Does the sample accurately reflect the characteristics of the intended population?**	**Is the choice of measurements suitable for the purpose or context?**	**Is there a low likelihood of non-response bias occurring?**	**Does the statistical analysis align with the research question and objectives?**	
Nigussie et al. (29)	No	No	Yes	Yes	Cannot tell	N/A
Yilma et al. (30)	No	No	Yes	Yes	Yes	Three
Patterson et al. (31)	No	No	Yes	Yes	Yes	Three
Marchant et al. (32)	Yes	Yes	Yes	No	Yes	Four
Dynes et al. (33)	No	No	Yes	Yes	Yes	Two
Karim et al. (34)	Yes	Yes	Yes	No	Yes	Four
Women & Children First (35)	No	No	No	Yes	Cannot tell	N/A
Avan et al. (36)	Yes	Yes	Yes	No	Yes	Four
Canavan et al. (37)	No	Yes	Yes	No	Yes	Three
Sibley et al. (38)	Yes	Yes	Yes	No	Yes	Four
Ayalew et al. (39)	Yes	Yes	No	No	No	Two

### Data synthesis and analysis

To ensure the effectiveness of our analysis protocol, we initially tested it on a small sample of studies to identify any potential issues or areas for improvement before proceeding with the full review. Additionally, addressing publication bias is a critical aspect of a systematic review, as it helps ensure that our findings are not influenced by selective reporting of studies. To achieve this, we employed comprehensive search strategies, which included utilizing multiple databases, exploring gray literature sources, examining conference proceedings, checking trial registries, and reaching out to experts in the field.

Given the considerable heterogeneity in QI methodologies, settings, and outcome measures, we opted not to conduct a meta-analysis. Instead, we employed a thematic data analysis framework to synthesize the data from the included studies. This approach involved organizing and presenting the data through text, tables, and figures. As a result, we summarized and tabulated the findings into three categories: (1) Information on overarching strategies for enhancing the quality of neonatal healthcare; (2) Information on the factors that either hindered or facilitated the implementation of QI interventions in neonatal healthcare; and (3) Information on the impacts of QI interventions on improving neonatal survival.

We categorized the findings based on the eight quality care standards outlined by the WHO and national quality care elements, including quality planning, quality control, and QI. Additionally, to synthesize the data, we employed a “vote counting method,” considering only the direction of the effect due to variations in effect measures and reported data across studies. Finally, we summarized and presented the results using the Donabedian quality of care framework, which includes the dimensions of structure, process, and outcomes.

## Results

### Search result

**Figure 1:**
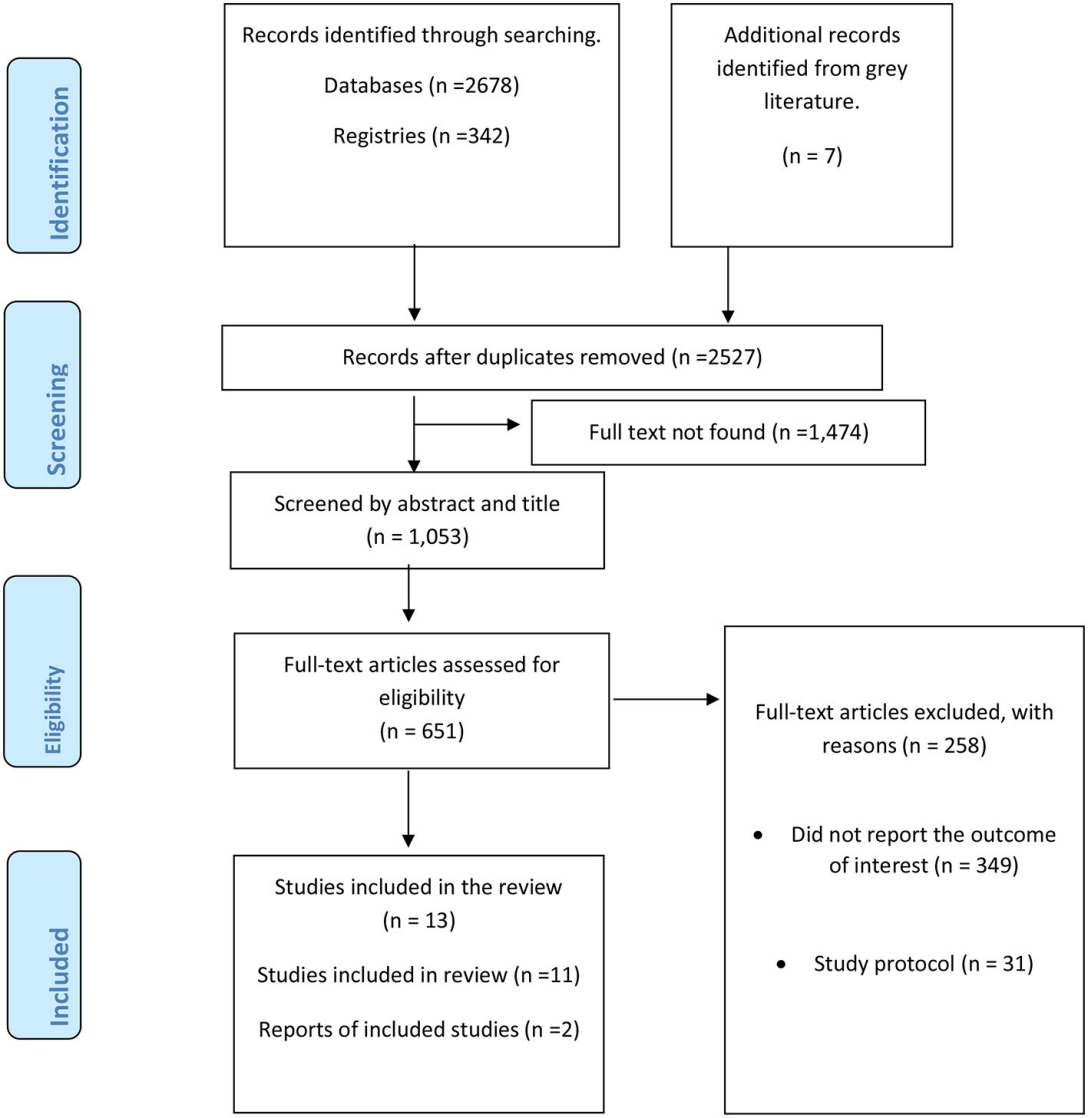
Preferred reporting items for systematic reviews and meta-analyses (PRISMA) flow diagram, Ethiopia, 2023.

The combined efforts of electronic and manual searches produced 3,027 articles. After eliminating duplicate reports, 2,527 articles were examined. One thousand fifty-three articles were retained after determining that the full text was unavailable for 1,474 articles. Afterward, we conducted a comprehensive assessment of 651 articles based on their full-text content. Ultimately, a total of 13 articles met the inclusion criteria (27–39). The PRISMA flow diagram outlines the stages of theme selection and provides the rationale for exclusions (see Figure 1).

### Study characteristics

The 13 studies in this analysis consisted of various types: one uncontrolled before and after study (38), ten project evaluation (29–37, 39), and two quasi-experimental studies (27, 28). These studies were conducted in Amhara, Tigray, Harari, Oromia, Addis Ababa city administration, and Southern Nation Nationality and People (SNNP) Regions. The analysis included a total of 821 health facilities across these studies. The participants involved in the studies were healthcare workers, health extension workers, health administrative staff, mother-infant pairs, Traditional Birth Attendants (TBAs), and community health volunteers. The main results derived from these studies were classified into QI outcome measures, different types of QI interventions, and factors that affect the implementation of QI interventions (Table 2).

**Table 2:** Summary of the study characteristics of the included studies, Ethiopia, 2023.

**References**	**Study type**	**Settings**	**Types of interventions**	**QI outcomes measure**	**Methods of interventions**	**Impacts of the interventions**
Nigussie et al. (29)	- Project evaluation	- SNNP, Amhara, Oromia & Tigray	- Mobile and electronic health interventions	- Timeliness, safety & effectiveness	- Health extension workers (HEW) register & prioritize newborns. - Providing automated job aids for HEWs. - Improving the referral and follow-up of neonatal healthcare services. - Utilizing mHealth applications to exchange information between health posts and health centers. - Sending SMS appointment reminders to clients. - Conducting training sessions. - Monitoring performance & tracing defaulters. - Implementing mHealth application-based supportive supervision.	- Enhanced communication among women, health posts, and health centers. - Implemented standardized neonatal healthcare services. - Promoted the use of qualified healthcare professionals for childbirth. - Enhanced postnatal care (PNC); achieved 83% improvement over the initial level.
Marchant et al. (32) and Yilma et al. (30)	- Project evaluation	- Oromia, Tigray, Amhara, SNNP, Harari & Addis Ababa	- Motivating the health care workers	- Safety, equity & effectiveness	- Recognizing high-performing HCWs through certificates and offering non-financial incentives to members of the health development army (HAD).	- Facility deliveries increased from 15% to 43%. - Delayed bathing increased from 39% to 50%. - Breastfeeding within the first hour increased from 50% to 66%. - PNC services showed improvement from 70% to 87%. - Referral rates were raised from 43% to 95%.
Canavan et al. (37), Marchant et al. (32), Yilma et al. (30), Patterson et al. (31), Avan et al. (36), Karim et al. (34), Sibley et al. (38), Women & Children First (35), Ayalew et al. (39), Hagaman et al. (28), and Hailemeskel et al. (27)	-Project evaluation, -Before/after study & -quasi-experimental	-Oromia, Tigray, Amhara, SNNP, Harari & Addis Ababa	-Training healthcare workers, -Upgrading infrastructure, -Implementing Integrated Community case management guidelines , -Providing necessary equipment and medical supplies & -Offering midwife-led continuity of care	-Safety, effectiveness, equity, efficient & patient-centered	-Provision of essential medicines and equipment. -Enhancement of the health information system. -Renovation of health centers and infrastructure. -Offering training and education programs for HEWs, healthcare workers, traditional birth attendants, and HDAs. -Conducting regular activities such as weekly updates, supportive supervision, mentorship, and monthly meetings for healthcare workers. - The intervention encompassed midwives working in partnership with other medical practitioners to offer immediate care throughout labor, delivery, and the postnatal phase.	-Strengthened referral systems between healthcare facilities. - Improved the quality of essential and immediate and PNC, including cord care with chlorhexidine, early breastfeeding initiation, thermal care, and immediate newborn assessment (Apgar). - Enhanced availability of essential medicines, clinical equipment, & vaccines. - Improved the ability of healthcare workers and health extension workers to manage serious diseases such as sepsis, pneumonia, malnutrition, and diarrhea. - Significant improvement in access to and equity in healthcare facility services.
- Increased availability of family health cards. - Improved neonatal survival. - Improved maternal perceptions regarding skilled delivery. - Increased rates of facility delivery and skilled birth care. - Improved access to and use of essential emergency obstetric and neonatal care. - Decreased occurrences of premature births, low Apgar scores, and admissions to neonatal intensive care units.
Marchant et al. (32), Yilma et al. (30), and Hagaman et al. (28)	-Quasi-experimental & project evaluation,	Oromia, Tigray, Amhara, SNNP, Harari & Addis Ababa	-Community mobilization and support	-Safety, people-centered, efficient, Equity & Effectiveness	-Engaging the HDAs to create connections between the community and the healthcare system. - Evaluating how the community perceives neonatal healthcare services and coordinating activities aimed at building team cohesion. - Encouraging cooperation between community health workers and volunteers to identify newborns in need of care and reinforcing partnerships between community members and primary healthcare facilities. -Raising community awareness through radio shows. -Encouraging experience sharing and peer-learning activities within the community.	-Significantly improved referral systems. - Increased coverage of PNC. - Increased adoption of vital and immediate newborn care practices, such as giving birth in healthcare facilities, delaying the baby's first bath, and initiating breastfeeding within 1 h of delivery. - Increased awareness among women to recognize newborn danger signs. - Enhanced knowledge of health workers regarding BEmONC - Improved maternal perceptions of giving birth in healthcare facilities. - Reduced risk of dangerous diarrhea in children and neonatal mortality. - Provision of appropriate treatment for sepsis. - Increased utilization of KMC

### Quality appraisal result

We evaluated the quality of the studies using the Mixed Methods Appraisal Tool (MMAT). Out of the 13 studies, 11 were subjected to a quality assessment. Among these, four studies received the highest quality rating, scoring a maximum of four on the MMAT scale. Five studies were considered moderately high quality, scoring three on the MMAT. Two studies were rated as having mediocre quality, receiving a score of two on the MMAT scale. Regrettably, two studies did not supply adequate information for a comprehensive MMAT assessment, as detailed in Table 1.

### Groups of quality improvements interventions outcome measures

The 13 studies examined QI interventions with a primary focus on delivering safe care with minimized risks and harm. Six studies sought to deliver sufficient care guided by scientific knowledge and evidence-based guidelines. Two studies targeted efficient care, emphasizing resource use and waste reduction. Only two studies specifically addressed the importance of timely, people-centered, and equitable care. Additionally, one study investigated multiple QI outcome measures, indicating a comprehensive approach.

### Category of quality interventions according to WHO standards

The interventions designed to enhance maternal and newborn care within healthcare facilities were classified according to the World Health Organization's (WHO) eight quality standard domains. The eight domains of WHO quality standards for improving the quality of maternal and newborn care in health facilities were used to categorize the quality interventions. It consists of evidence-based practice of routine care and complications management, actionable information systems, functional referral systems, effective communication, respect and preservation of dignity, emotional support, competent, motivated human resources, and essential physical resources available, respectively (13).

Most of these interventions concentrated on the first domain, which deals with the evidence-based routine upkeep and handling of complications. Furthermore, domain seven, which revolves around ensuring the competency of staff, emerged as a frequent target for intervention. Some interventions were directed toward domains three and eight. However, there were no interventions identified that specifically addressed domains 2, 4, 5, and 6.

### Category of interventions according to the three core elements of quality

QI interventions that aim to enhance the quality of neonatal healthcare services can be classified into three categories: planning, control, and quality improvement. These categories align with the three national core elements of quality (40). Some focused-on planning (*n* = 3) (30, 34, 39) and control (*n* = 4) (27, 28, 30, 37), while most focused-on quality planning (*n* = 12) (27–33, 35–39).

#### QI interventions focused on quality planning

Quality planning is crucial for providing the proper care to patients at the right time (40). It involves distributing resources, such as essential medicine and equipment, and improving infrastructure (30, 34, 35, 39). The interventions that targeted quality planning resulted in several positive outcomes. These included increased availability and utilization of essential and immediate newborn care, skilled birth care, delayed bathing, postnatal care (PNC), and prompt newborn assessment. Moreover, enhancements were observed in the availability of medical supplies and equipment. These interventions also contributed to a reduction in neonatal mortality, increased maternal perceptions and awareness about neonatal danger sign, and enhanced referral and feedback systems between healthcare facilities (30, 35, 39). Furthermore, there was a decrease in the risk of dangerous diarrhea. Interventions such as the construction of health posts had a significant impact on improving access and equity to healthcare facilities (34).

#### Interventions focused on quality control

Quality assurance or control is a regulatory procedure designed to uphold or enhance the quality of care by minimizing errors (40). In densely populated regions of Ethiopia, several initiatives were put into action to enhance the accessibility and quality of neonatal healthcare services. These programs included the Strengthening Ethiopian Urban Health Extension Program (SEUHEP), Community Maternal and Neonatal Health (CMNH) program, the last 10-kilometer project, and the Standard Based Management and Recognition (SBM-R) program initiated by JHPIEGO (28, 30, 35, 37). These interventions significantly improved the mean quality scores, including basic infrastructures, emergency obstetrics, neonatal care, pediatrics care, laboratory services, guidelines and auditing, infection protection, and patient safety. The average quality score rose from an initial value of 65.6 ± 10.5 to a final score of 91.2 ± 12.4 (37). Consequently, there was a 33% reduction in neonatal mortality, improved maternal perception of institutional delivery, and a decreased risk of dangerous diarrhea among children (30, 35). Additionally, interventions involved strengthening health information systems, midwifery lead continuity care, promoting referrals, supporting facility leadership, identifying issues, and developing testable solutions to address gaps in care. These interventions result in referral systems between health institutions were strengthened, and the management of sepsis improved from 87.5% to 99%. Furthermore, there was a reduction in the number of premature births, instances of low Apgar scores at 5 min after birth, and admissions to neonatal intensive care units (28).

#### Interventions focused on quality improvement

QI interventions entail a collaborative effort involving healthcare providers, patients, families, researchers, payers, planners, and educators with the aim of improving patient outcomes, performance, and professional development (40). Numerous interventions have been put into practice to enhance neonatal healthcare services. These interventions are geared toward fortifying healthcare systems, which remains a fundamental pillar in achieving the Millennium Development Goals and will continue to play a pivotal role in reaching the Sustainable Development Goals (41). Such interventions encompass mobile and electronic health strategies (mHealth), training and education initiatives, mentoring programs, regular meetings, non-financial incentives, community-based support for newborn care, and peer-based support programs (28–33, 35–39, 42). mHealth interventions utilize wireless technologies to enhance skilled delivery, standardized neonatal healthcare, and referral systems (43–45). However, the impact of mHealth interventions on neonatal healthcare services requires further investigation. QI interventions, such as community mobilization, training in community-based newborn care, and partnerships between community actors and primary health facilities, have successfully enhanced access and utilization of neonatal healthcare services. These interventions have led to increased facility delivery rates, delayed bathing, breastfeeding initiation, and utilization of clinical equipment (30, 32). Additionally, supportive supervision and training of healthcare workers in essential newborn care have positively influenced immediate newborn care practices and improved neonatal health outcomes implementing interventions involving healthcare worker training and deployment has improved access and equity in health facilities (31). However, disparities remain in postnatal care visits, with fewer visits to poor women. Overall, these interventions have shown positive effects on newborn care but have had limited impact on care-seeking behaviors for childhood illnesses and vaccination rates among specific population groups (36).

### Donabedian quality of care framework

We employed the Donabedian quality of care framework to examine and present the findings of this review because it is a widely accepted and suitable framework for this purpose. As a result, the quality indicators utilized to evaluate the quality of care in the studies included in this review were divided into structure, process, and outcomes. Most of the studies primarily focused on quality measures related to the process (*n* = 12) (27–35, 37–39), while some also considered aspects related to structure (*n* = 6) (30, 33–35, 38, 39) and outcomes (*n* = 6) (27, 28, 31, 33, 35, 36) (Table 3).

**Table 3:** Donabedian quality of care framework, Ethiopia, 2023.

**Study characteristics**		**Outcomes according to Donabedian framework**								
**References**	**Interventions**	**Process**			**Structure**			**Outcomes**		
		**Immediate and essential newborn care**	**Infection prevention and patient safety**	**Referral & feedback b/n health center and health posts**	**Supplies, medicine, equipment & infrastructure**	**Service availability**	**Guidelines**	**Neonatal mortality & morbidity**	**Recognizing neonatal danger sign**	**Knowledge of HEWs about CBNC**
Canavan et al. (37)	- Providing training & the provision of medical equipment	-Increased	-Increased	-	-Increased	-Increased	-Increased	-	-	-
Nigussie et al. (29)	-mHealth interventions	-Increased	-	-Increased	-	-	-	-	-	-
Yilma et al. (30)	- Distributing file folders for referral and feedback records, arranging weekly updates and monthly gatherings for HCWs and HEWs, engaging Health Development Armies (HDAs) to create links between the community and the healthcare system, delivering technical assistance for QI Teams (QITs), providing non-monetary incentives, bolstering the Health Management Information System (HMIS), establishing connections between health centers and health posts, extending operational hours and services, and furnishing essential equipment and supplies.	-	-	-Increased	-Increased	-	-	-	-	-
Patterson et al. (31)	- Providing training for HCWs on immediate and essential neonatal care, as well as complication management.	-Increased	-	-	-	-	-	-Decreased	-	-
Avan et al. (36)	-Providing training on CBNC and ensuring the availability of drugs, medical equipment, and infrastructure.	-	-	-	-Increased	-	-	-	-	-Increased
Dynes et al. (33)	-Providing training about CBNC	-Increased	-Increased	-	-	-	-Increased	-	-Increased	-Increased
Marchant et al. (32)	- Actively involving community health care workers, HDAs, and volunteers in the healthcare systems. -Training and non-financial incentives were provided specifically for the HDAs.	-Increased	-	-	-Increased	-	-	-	-	-
Karim et al. (34)	-Health center & infrastructure renovations.	-Increased	-	-	-Increased	-	-	-	-	-
Sibley et al. (38)	-Providing training about CBNC	-Increased	-	-	-	-	-	-	-	-
Women & Children First (35)	-Offering training for health workers and health volunteers, implementing peer-based interventions and/or support programs, supporting referral systems, providing essential equipment and drugs, and conducting supportive supervision	-Increased	-	-	-	-	-	-Decreased	-Increased	-Increased
Ayalew et al. (39)	-Providing training on standard-based management and recognition & on BEmONC, supplying essential supplies and equipment, & conducting regular follow-up activities	-Increased	No significant difference	-	-Increased	-	-	-	-	-
Hagaman et al. (28)		-Increased	-	-	-	-	-	-Decreased	-	-
Hailemeskel, et al. (27)	- Offering midwife-led continuity of care	-Increased	-	-		-	-	-Increased	-	-

### Barriers and facilitators to implement QI interventions

The studies pinpointed factors that can either support or impede QI interventions, as outlined in Table 4. In total, 20 obstacles were documented, with 15 occurring at the local level and 5 at the system level. Furthermore, 14 enhancers were recognized, with 10 operating at the local level and 4 at the system level. It's worth noting that the statistical significance of these enhancers and obstacles was not evaluated.

**Table 4:** Factors affecting the efficacy of QI measures, Ethiopia, 2023.

**Local level**	**References**	**System level**	**References**
**Facilitators**			
- Precise and well-defined objectives for improving quality	(30)	-The commitment of regional health bureau's involvement in and buy-in of the intervention;	(30)
-Motivated health extension professionals;		-National hospital performance management efforts;	(37)
-The commitment of the health center management;	(37)	-Fully-scaled government &	
-Smooth handover;		-Stakeholder engagement	
-The linking of performance to financial rewards for top performing and most improved hospitals			
-Sustain needed resource;			
-Iterating implementation based on experience;			
-Frequent and consistent QIteam meeting;			
-Pre-intervention need assessment &	(29)		
-ICT-supported guidance tool;			
**Barriers**			
-Deficient in staff knowledge and practice;	(29, 37)	- Limited engagement and support from THOs	(30)
-Constrains of time;	(29, 31)	Limited engagement and support from regional health bureau;	
-Shortage of critical infrastructures	(37)	- Limited integration of the QIT with the programmers	
-Incomplete charts;		Lack of ownership of QI initiatives by QIT &	
-Using clinical observations as quality evaluations methods;		- Insufficient funds.	(37)
-Using lead hospitals as populations;			
- Multiple QI measures	(29)		
-Delayed distribution of equipment;			
-Poor monitoring of the service delivery;			
-Weak internet signal;			
-Protracted and widespread internet connectivity interruption;			
-Failure in timely reporting of non-functional device;			
-Delayed replacement of damaged or lost resources;			
-Lack of efficient and sufficient equipment;			
-High turnover of trained workforce;			
-Regarding community-based intervention as an added duty for healthcare personne	(30)		
-In adequate documentation and data use;			
- Insufficient healthcare personnel at the health center			
- Restricted involvement and assistance from the management of health centers, and			
-Loose linkage between health center staffs and community health workers (HEWs)			

### Facilitators

Three studies identified ten facilitators at the local level. These include having well-defined and targeted QI objectives, motivated health extension professionals, dedicated health centre management, linking performance to financial incentives, conducting pre-intervention needs assessments, utilizing information and communication technology (ICT) tools for guidance, adjusting implementation based on experience, maintaining essential resources, ensuring a seamless handover, and conducting regular and consistent QI team meetings (29, 30, 37). Additionally, two studies identified four facilitators at the system level, which encompassed stakeholder engagement, the full-scale involvement of the government, national hospital performance management initiatives, and the commitment and support of regional health bureaus in implementing and endorsing the intervention (30, 37).

### Barriers

Similarly, four studies identified 20 barriers that locally hindered the implementation of QI initiatives. These barriers include insufficient staff knowledge and practice, time constraints, relying solely on lead hospitals as the target population, scarcity of critical infrastructures, utilizing clinical observations as the primary method for evaluating quality, the use of multiple QI measures, delays in equipment distribution, inadequate monitoring of service delivery, weak internet signal, prolonged and widespread connectivity interruptions, failure to report non-functional devices in a timely manner, and delays in replacing damaged or lost resources. In addition lack of efficient and sufficient equipment, high turnover of trained workforce, considering community-based interventions as an additional burden for health staff, inadequate documentation, and data utilization, insufficient staff numbers in health centers to support and implement QI initiatives, limited engagement and support from health center management, and weak linkages between health center staff and community health worker were hinder the quality of care (29–31, 37).

In two studies, five obstacles were identified at the system level. These barriers encompassed insufficient funding, limited involvement and backing from the District Health Offices, restricted engagement and support from the regional health bureau, inadequate integration of the QI teams with existing programs, and a deficiency in the sense of ownership of QI initiatives by the QI team (30, 37).

## Discussion

### Evidence gaps and research priorities

This indicates the first systematic review of interventions aimed at enhancing neonatal healthcare quality in Ethiopia. In this context, we identified thirteen articles, the majority of which described interventions targeted at healthcare teams and healthcare services. The WHO advocates for a more comprehensive integration of the three fundamental components of quality care, which encompass quality planning, quality control, and QI (13). It's essential to emphasize that improving the quality of care necessitates adequate focus not only on QI interventions but also on quality planning and control. Additionally, it should be important to develop QI model to improve access to quality neonatal health care service and neonatal survival in Ethiopia.

The significance of healthcare workers, infrastructure, equipment, and medical supplies in improving the quality of healthcare systems is emphasized by various interventions such as training, upgrading, and the provision of supplies. This aligns with findings from previous reviews of maternal and neonatal health initiatives in the Pacific region and sub-Saharan Africa (46, 47). It's crucial to recognize that a high-quality healthcare system cannot exist without an adequate healthcare workforce and proper infrastructure, equipment, and medical supplies. Therefore, it is essential to empower, train, and support the healthcare workforce to ensure they can provide quality care and effective coverage. Achieving this requires investments in infrastructure, as well as ensuring accessibility, acceptability, and the availability of high-quality equipment and medical supplies. However, many low- and middle-income countries, including Ethiopia, face challenges related to staff training, infrastructure, equipment, and medical supply shortages. Programs addressing these shortages for various groups of healthcare workers have limited impact on healthcare services (48). Sustainable training and education programs conducted at universities and training colleges can have far-reaching benefits, particularly if they include leadership and management skills (49).

Several interventions have focused on training volunteers, such as Traditional Birth Attendants (TBAs) and Health Development Armies (HDAs), to serve as village birth attendants, addressing gaps in areas with a shortage of trained healthcare workers (32, 33, 38). However, relying solely on volunteers, who are often motivated by willingness, may not be a sustainable long-term solution (50). Therefore, formal recognition, compensation, and access to formal training are measures that can improve the motivation of volunteers (51). For project and program designers, investing in in-service training and education should be a primary recommendation for QI initiatives. Additionally, Health Extension Workers have played a vital role in enhancing the quality of neonatal healthcare services in Ethiopia (52). However, their effectiveness is hindered by a lack of skills and motivation (53). Therefore, offering fair compensation and incentives to Health Extension Workers can help motivate and retain skilled professionals (54).

Despite the limited available data regarding the impact of mHealth interventions on the quality of neonatal healthcare services and neonatal health outcomes in Ethiopia, it's worth noting that mHealth interventions represent an innovative approach to enhancing the quality of neonatal healthcare services. mHealth interventions often involve wireless, portable information, and communication technologies such as phones, computers, personal digital assistants, and digital point-of-care testing devices to support health and health care. It also comprises two communications between the health care system and clients through the provision of SMS-based appointment notification messages for the client, providing information for HEWs about clients who received service at health centers, and strengthening referral and referral feedback (43–45). Existing evidence suggests that mHealth interventions contribute to improved quality of neonatal care and neonatal survival rates (55). Furthermore, there is insufficient data assessing the effectiveness of community mobilization and peer-based interventions in this context. Consequently, further research is needed to evaluate the impact of these interventions on the quality of neonatal healthcare in Ethiopia. This will help inform evidence-based recommendations for enhancing policies, programs, and educational initiatives.

While providing financial incentives to beneficiaries and rewarding healthcare workers (HCWs) for improved performance has demonstrated its potential to enhance neonatal survival rates in Ethiopia (56, 57), there is a lack of interventions focused on financial incentives. It is well-documented that quality of care is of vital importance for underserved and disadvantaged populations. However, there is a shortage of evidence concerning QI initiatives that specifically lead to improvements in the uptake of quality services by the poorest quintile. Additionally, studies that concentrate on neonatal health-specific interventions, such as neonatal and perinatal mortality audits, require robust and standardized data collection mechanisms to effectively assess their effectiveness. Therefore, enhancing health information systems in countries is imperative to evaluate the impacts of mortality audits.

### QI interventions based on national outcome measures

Quality of care revolves around the delivery of healthcare services that are safe, effective, timely, efficient, equitable, integrated, and centered around the needs of patients (14, 15). However, it's important to note that only a subset of the studies in this review provided information on quality measures related to efficiency, patient-centeredness, equity, and timeliness of care. Therefore, there is a need for further research to identify proven interventions that can enhance these aspects of quality.

Additionally, it's crucial to recognize that the five regions included in this review are characterized by cultural and linguistic diversity, with each region having its own unique cultural beliefs, values, and customs that significantly influence neonatal health practices (58–60). Surprisingly, none of the QI interventions included in this review took socio-cultural considerations into account in their approach. Socio-cultural factors have a substantial impact on the acceptability of healthcare services for both patients and their families. Therefore, integrating socio-cultural considerations should be a fundamental component in the design and implementation of any QI interventions (61).

### Quality interventions according to WHO standards

The health system approach to QI interventions encompasses two integrated dimensions: care provision and care experience. Care provision involves the use of evidence-based practices for routine care and the management of complications, actionable information systems, and functional referral systems. On the other hand, care experience pertains to effective communication regarding the care provided, respect for and preservation of dignity, as well as emotional support. These dimensions are underpinned by cross-cutting domains of quality of care, which encompass the availability of competent and motivated human resources and adequate physical resources (13).

In the context of the reviewed interventions, many of them primarily targeted Domain 1 (the utilization of evidence-based practices for routine care and complication management) and Domain 7 (ensuring the availability of competent and motivated human resources). Conversely, very few interventions were directed toward Domain 3 (establishing functional referral systems) and Domain 8 (ensuring the availability of essential physical resources). Notably, there were no interventions focused on Domain 2 (implementing an actionable information system) or the aspects related to the experience of care received. It's important to recognize that integrating care provision and care experience within the domain of quality care is crucial in QI initiatives. The absence of respectful and dignified care, advanced information systems, and effective communication with clients and their families is a global issue, but it is particularly challenging in Low-and Middle-Income Countries (LMICs) that are strained and under-resourced (62). Insufficient attention to these aspects can lead to disrespectful or abusive healthcare experiences, discouraging clients from seeking such services in the future and increasing the risk of adverse outcomes (20). Therefore, improving the quality of care requires equal attention to both the provision and experience of care, alongside other domains of quality of care.

Implementing standardized guidelines and protocols for neonatal healthcare can promote a systematic approach to assess and enhance the quality of neonatal healthcare services. However, there is a need for more evidence regarding the impact of interventions aimed at implementing updated and standardized guidelines and protocols for neonatal healthcare services and their effects on health outcomes. Additionally, it is essential to conduct studies on the social acceptability and long-term sustainability of QI initiatives. Therefore, future research should prioritize investigating the social acceptability of QI initiatives using robust study designs. In this review, only a few of the QI interventions were based on standardized quality indicators. Hence, there is a need for further research that employs standardized quality of care indicators as a basis for evaluating QI initiatives.

### Barriers and promoters of QI interventions

Although there is limited evidence available on the obstacles and drivers of QI interventions, identifying these barriers and promoters for successful QI initiatives is crucial for expediting progress toward achieving quality-of-care goals. Therefore, healthcare planners should consider addressing obstacles such as staff shortages, financial constraints, infrastructure limitations, and time constraints, which may become more pronounced during periods of increased seasonal demand. Additionally, offering financial incentives to leading hospitals to support their projects can be a viable strategy.

It's important to note that none of the studies assessed the statistical significance of these barriers and promoters. Conducting multi-center studies could enable a more in-depth analysis in this regard. Furthermore, the lack of skilled, competent, motivated, and dedicated healthcare workers poses a significant barrier to improving the quality of neonatal healthcare services. Therefore, a proactive approach would involve enhancing the skills, competency, and motivation of healthcare workers through various short- and long-term training programs.

In general, there is a wide array of innovative interventions being implemented globally to enhance the quality of neonatal healthcare services. Consequently, there is a pressing need for comprehensive evidence to evaluate the most effective combinations of QI initiatives. This requires collaborative efforts among researchers, stakeholders, governmental bodies, and non-governmental organizations to formulate policies and healthcare models that align with the specific needs of their respective populations.

Additionally, it is imperative to design approaches that empower healthcare providers, whether in the community or at healthcare facilities, and program managers at the district level to adopt and implement patient-centered, evidence-based interventions aimed at improving the quality of neonatal healthcare. Furthermore, assessing the impact of various interventions on the quality of care and neonatal health outcomes in Ethiopia was challenging due to the absence of statistical significance in the studies. Therefore, these interventions would benefit from more extensive, large-scale studies or more rigorous evaluation processes.

Moreover, there is a need for more evidence concerning the sustainability and scalability of these interventions, particularly in resource-constrained settings with fragile healthcare systems. Future research should delve into the factors influencing the sustainability of interventions when scaled up and assess their cost-effectiveness. Furthermore, it is essential to conduct in-depth research on how the most impactful interventions for enhancing quality of care are being implemented across various contexts and settings.

Finally, despite the observed improvement in neonatal healthcare services, as indicated by the pooled results of this study, it is worth noting that there are still healthcare facilities facing shortages of essential medicines, equipment, and basic neonatal healthcare services. This discrepancy may be attributed to the fact that most of the included studies assessed the effectiveness of specific projects. Therefore, it is recommended that future researchers in this field conduct comprehensive, multi-setting surveys to accurately gauge the status of national neonatal healthcare services.

### Limitations of the study

This study provides a comprehensive overview of QI interventions in Ethiopia, drawing from national data. However, it's important to recognize certain limitations for future research. Firstly, there's a need for studies from five specific regions in Ethiopia, and some of the available studies had small sample sizes. The review relied solely on published literature, excluding unpublished QI initiatives. Furthermore, the included studies described interventions and outcomes but didn't assess their actual effectiveness. There was significant variability in terms of study populations, QI interventions, and outcome measures, leading to heterogeneity challenges. The study also didn't consider parental experiences, a crucial aspect of improving quality care.

Many of the primary studies were conducted within project-specific contexts, limiting the generalizability of their findings. This context-specific nature could introduce biases like researcher bias, selection bias, and response bias, potentially compromising result reliability. Practical challenges, such as limited access to control groups and reliable outcome measures, could also affect the findings. Additionally, the long-term impacts and sustainability of the QI interventions weren't evaluated. Therefore, it's important to note that the review might not fully represent the entire landscape of QI interventions in Ethiopia. Caution is advised when interpreting and applying these findings, considering the inherent limitations of the primary studies and the current analysis.

However, by recognizing these limitations, this review offers valuable insights for shaping future studies that aim to enhance the quality and reliability of research in this field.

## Conclusions

Even though Ethiopia's newborn health care systems are not exceptionally good, most QI initiatives there concentrate on improving the health care systems. Therefore, the ongoing and prospective QI interventions should place equal emphasis on mHealth interventions, financial incentives, community mobilization, provision, and experience of care as they do on improving the health care systems. Furthermore, it is crucial that QI initiatives prioritize the delivery of healthcare that is safe, effective, timely, efficient, equitable, integrated, and patient centered. Nevertheless, challenges such as limited resources, inadequate infrastructure, and shortages of medical supplies can hinder the effectiveness of QI interventions. Consequently, improving quality in Ethiopia demands sustained and substantial investments in terms of efforts, resources, and technical expertise. These investments are essential for bolstering the healthcare workforce, enhancing infrastructure, and ensuring the availability of necessary supplies. This, in turn, will support the successful implementation of research into QI initiatives. This requires leadership commitment for quality planning, improvement, and control. Advocacy is also crucial toward better quality of care. Moreover, continued training and capacity building programs for health care workers should be prioritized. Consistent and encouraging oversight and guidance provided by more experienced healthcare practitioners can enhance the competence and expertise of those at lower levels of the healthcare hierarchy. Additionally, reinforcing cooperation among healthcare personnel is of paramount importance. Expanding community involvement efforts, including initiatives like raising awareness, conducting health education sessions, and engaging local leaders and traditional birth attendants, can also serve to encourage behaviors that promote neonatal health.

## Data availability statement

The original contributions presented in the study are included in the article/Supplementary material, further inquiries can be directed to the corresponding author.

## Author contributions

DB: Methodology, Supervision, Validation, Writing – original draft, Writing – review & editing. DE: Conceptualization, Writing – review & editing. WB: Writing – review & editing. YK: Writing – review & editing. BM: Conceptualization, Methodology, Validation, Writing – review & editing. YA: Conceptualization, Data curation, Investigation, Methodology, Supervision, Validation, Visualization, Writing – review & editing.
