# Supplementary material for: Improving the quality of neonatal health care in Ethiopia: a systematic review
Source: Front Med (Lausanne). 2024 May 22;11:1293473. doi: 10.3389/fmed.2024.1293473 (PMC11150606; doi:10.3389/fmed.2024.1293473)
Supplement: Supplementary file 2 [file Data_Sheet_1.docx]

| Data base | Search Number | Query | Results |
| --- | --- | --- | --- |
| PubMed | #4 | **(((((Quality) OR (quality improvement)) OR (quality indicator)) AND (Ethiopia)) AND (((interventions) OR (approaches)) AND (Ethiopia))) AND ((((((((((postnatal care) OR (infant care)) OR (perinatal care)) OR (child health service)) OR (health care)) OR (health services)) OR (neonatal care)) OR (newborn health)) OR (maternal-child health service)) AND (Ethiopia))** | 1,616 |
|  | #3 | **(((((((((postnatal care) OR (infant care)) OR (perinatal care)) OR (child health service)) OR (health care)) OR (health services)) OR (neonatal care)) OR (newborn health)) OR (maternal-child health service)) AND (Ethiopia)** | 12,664 |
|  | #2 | **((interventions) OR (approaches)) AND (Ethiopia)** | 18,922 |
|  | #1 | **(((Quality) OR (quality improvement)) OR (quality indicator)) AND (Ethiopia)** | 3,583 |

Additional file 2: Search strategy for PubMed database
